# Spontaneous Inflammation of the Alveolo-Dental Membrane

**Published:** 1853-10

**Authors:** Chapin A. Harris


### ARTICLE IX.

### *Spontaneous Inflammation of the Alveolo-dental Membrane.*

By Chapin A. Harris, M. D., D. D. S.

About three years since, Miss T., a maiden lady, thirty-five years of age, of a scrofulous habit, applied to me to extract a lower molar, which had been the seat of severe pain for some six or eight weeks. Perceiving, on exmaination, that the crown of the tooth was sound, I recommended the application of a leech to the gum. This did not mitigate the pain in the slightest degree. 'As the crown of the tooth was free from caries, and the character of the pain did not indicate inflammation of the pulp, I suspected it arose from the action of some constitutional cause, and advised her to consult her medical attendant before submitting to the operation of extraction. She followed my advice, but before the treatment which he instituted had produced any effect, the pain became so intense, she called upon me again, and this time, at her earnest solicitation, I removed the tooth. The roots, on examination, were found to be covered with thin blood, of a dark purple color, which had seemingly been effused through the coats of the small capillary arteries distributed upon the periosteum.

A few weeks after the removal of this tooth, I was requested to extract the corresponding molar on the other side in the same jaw, and under precisely similar circumstances. I again advised the application of a leech, and such other constitutional treatment as the state of her general health, might, in the opinion of her medical adviser, seem to indicate. But as she had already suffered severe pain from it for more than two weeks, I could not persuade her to have the operation delayed. The roots of this tooth presented the same appearance as those of the other.

Seven or eight weeks after the last operation, she visited me again. Two other teeth, an upper molar and a lower bicuspid, had become the seat of constant, gnawing pain. Both of these teeth were slightly affected with caries, but the structural alteration had penetrated but a short distance into the dentine, and could have had no agency in the production of the pain, which, as in the two former cases, was evidently the result of periodontitis, and that not caused by any other source of local irritation than the mere presence of the teeth, but dependent upon great preternatural irritability of the periosteum, arising from some peculiar cachectic habit of body, or state of the general health. Entertaining this view of the case, and not wishing to interfere with the general treatment which seemed evidently to be indicated, I advised her to have leeches applied to the gums of the affected teeth, and to place herself under the care of her physician, to whom, at her request, I addressed a note, expressing my opinion with regard to the cause of the pain from which she was suffering. As she resided in the country, some twelve or fifteen miles from Baltimore, I had great difficulty in persuading her to return with the aching teeth in her mouth, but yielding to my solicitations, she finally consented to do so. She returned immediately, sent for her physician, and was at once put under medical treatment, which was perseveringly continued for about seven weeks. During this time, aperients, tonics, such as quinine and the various preparations of iron, counter irritants and narcotics were prescribed; but the pain continued without any mitigation, and in the meantime, extended to two of her other teeth. It had become so agonizing, that she was unable to obtain any sleep at night, except when under the influence of large doses of morphia, and despairing of relief, she again visited the city, firmly resolved to have the four aching teeth removed. Her suffering was now so great, that I no longer refused to perform the operation. The roots of these teeth presented the same appearance as those of the two first.

Miss T. left Baltimore, the day after the operation, comparatively free from pain; but the sockets remained sore, and at times, slightly painful for several weeks.

About three months after the removal of the last teeth, another began to ache, and in about three weeks, the pain having assumed such a degree of severity as to render its longer endurance almost insupportable, she came to the city and had the tooth extracted. The loss of this procured a few weeks freedom from pain; but in a short time, another was seized, and was ultimately removed. In this way, tooth after tooth was attacked and extracted, until at the expiration of about eighteen or twenty months, all of the molars and bicuspids, except one, of both jaws, were removed.

Believing that the extreme irritability of the alveolo-dental periosteum, which seemed so great, that the mere presence of the teeth acted as irritants, arose, principally, from a scrofulous diathesis of the general system, I suggested the use of iodide of potassium. This was tried, beginning with two drops a day of Lugol's solution. The dose was gradually increased, until the whole system had become, as it were, completely saturated with it, but with no better effect than the remedies which had been previously prescribed. The inflammation soon extended to the sockets of the remaining teeth, attended by the most agonizing pain, and one after another was removed, until not a single tooth remained in either jaw.

The roots of all the teeth presented the same appearance, and what seemed very remarkable, the inflammation at no time extended to the gums; this structure exhibited no indication of increased vascular action, but retained throughout the whole progress of the disease, a pale, bluish-rose-colored tinge; their margins were thin and regularly festooned. The pulps of the teeth were also free from inflammation, and the hard structures of the organs were, for the most part, free from caries. Some six or eight were slightly affected, and four had been filled, but in no instance, had the disease extended to the pulp-cavity.

Up to the time of the development of this most singular affection, the patient had lost but six teeth; the remainder, twenty-six in number, were removed in a little more than two years.

